# Carboxypeptidase Y activity and maintenance is modulated by a large helical structure

**DOI:** 10.1002/2211-5463.12686

**Published:** 2019-06-17

**Authors:** Mai Makino, Takehiko Sahara, Naoki Morita, Hiroshi Ueno

**Affiliations:** ^1^ Department of Biochemistry Nara Medical University Kashihara Japan; ^2^ Bio‐Design Research Group, Bioproduction Research Institute National Institute of Advanced Industrial Science and Technology (AIST) Tsukuba Japan; ^3^ Molecular and Biological Technology Research Group, Bioproduction Research Institute National Institute of Advanced Industrial Science and Technology (AIST) Sapporo Japan; ^4^ Laboratory of Biochemistry and Applied Microbiology, School of Agriculture Ryukoku University Otsu Japan

**Keywords:** disulfide bond, protein folding, serine carboxypeptidase, α/β hydrolase fold

## Abstract

Yeast carboxypeptidase Y (CPY) is a serine protease with broad substrate specificity. Structurally, CPY belongs to the α/β hydrolase fold family and contains characteristic large helices, termed the V‐shape helix, above the active site cavity. Four intramolecular disulfide bonds are located in and around the V‐shape helix. In this study, mutant CPYs were constructed in which one of these disulfide bonds was disrupted. Mutants lacking the C193–C207 bond located at the beginning of the V‐shape helix aggregated easily, while mutants lacking the C262–C268 bond located at the end of the V‐shape helix displayed decreased hydrolytic activity. The results indicate that the V‐shape helix is involved in CPY catalysis and in maintenance of its conformation.

**Abbreviations**
CPWII: wheat carboxypeptidase II
CPY: carboxypeptidase Y
ERAD: endoplasmic reticulum‐associated degradation
mCPY: mature CPY
PPCA: protective protein/cathepsin A
pro‐CPY: precursor CPY

Carboxypeptidase Y (CPY) is a vacuolar serine‐type protease isolated from yeast, *Saccharomyces cerevisiae*. CPY undergoes several maturation steps to form mature CPY (mCPY) that exhibits exopeptidase activity toward peptides, esters, amides, and anilides with broad substrate specificity 1, 2, 3, 4. mCPY has been crystallized, and its three‐dimensional structure was determined 5. Analysis of the structural anatomy of CPY using computer graphics revealed that mCPY has a typical α/β hydrolase fold pattern with two domains: a core domain (1–179 and 318–421) and an ‘insertion’ domain (180–317; Fig. 1A). The topology of the core domain of CPY is similar to that of the canonical core of the α/β hydrolase fold family 6 (Fig. 1B). The residues that form the catalytic triad, S146, H397, and D338, are all located within the core domain. The insertion domain contains helices that are attached to the conserved core folding topology (Fig. 1B). This helical insertion topology is often found in the α/β hydrolase fold family. Each insertion may have unique roles: For example, an insertion domain acts as a helical lid in most lipases where it helps control the access of substrate to the active site by blocking the entry of solvent to shield the active site pocket 7, 8; three insertions in acetylcholinesterase are proposed to be involved in channeling the substrates to the active site 9, 10.

**Figure 1:**
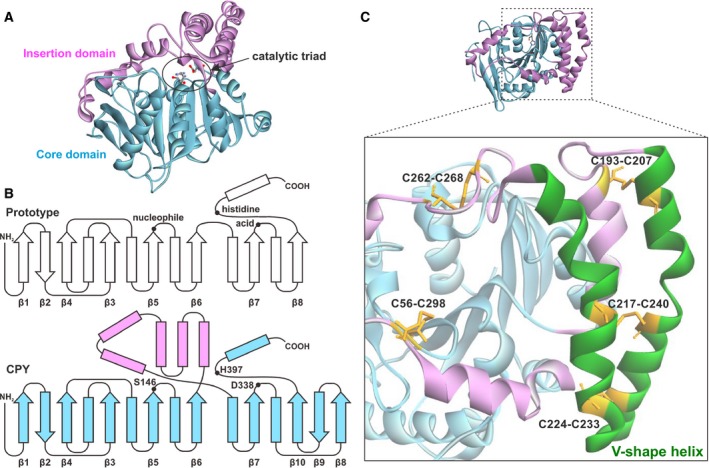
(A) Overall structure of mCPY (PDB ID: 1YSC). The core domain (1–179 and 318–421) is colored light blue, and the insertion domain (180–317) is colored pink. The catalytic triad (S146, H397, and D338) is located within the active site cavity of the core domain. (B) Schematic diagram of the topology of the α/β hydrolase fold. Arrows and rectangles denote β‐strands and α‐helices, respectively. Dots indicate the nucleophile, acid, and histidine residues of the catalytic triad. In a prototypic hydrolase fold pattern, a central core is composed of eight β‐strands mixed with α‐helices. In CPY, the large structure colored in pink is inserted between β‐strand 6 and the following α‐helix, designated the ‘insertion domain’. (C) Location of the V‐shape helix and intramolecular disulfide bonds in CPY structure. The V‐shape helix (204–251, colored green) consists of two large helices, which are a part of the insertion domain and appear to lie over the active site cavity. Cysteine residues forming disulfide bonds are shown as the orange stick. Four out of five disulfide bonds are located around the V‐shape helix.

As a part of the insertion domain, CPY has two adjacent large α‐helices, collectively termed the ‘V‐shape helix’. Examination of the crystal structure of mCPY has revealed that the V‐shape helix juts out from the core domain, reminiscent of a bird wing. The V‐shape helix appears to cover the active site cavity. Structurally, the V‐shape helix features four intramolecular disulfide bonds (Fig. 1C). Two disulfide bonds are located near the beginning (C193–C207) and the end (C262–C268) of the V‐shape helix. The two other disulfide bonds, C217–C240 and C224–C233, bridge two α‐helices that form the V‐shape helix 11. The absence of hydrogen bonds between the V‐shape helix and the core domain supports the view that the V‐shape helix plays a role as a flexible gate for the entry of substrate into the binding pocket. A similar V‐shape helix is present in homologous serine carboxypeptidases, including wheat carboxypeptidase II (CPWII) and human protective protein/cathepsin A (PPCA), although the sequence similarity is low 12, 13. Based on these considerations, it is reasonable to suggest that the V‐shape helix is a common motif among serine carboxypeptidases that contain an α/β hydrolase fold and that the helix plays significant role(s) in catalytic activities.

In this study, we investigated whether or not the V‐shape helix is involved in the activity of CPY by constructing mutant CPYs in which the C193–C207 or C262–C268 disulfide bonds present in the V‐shape helix were disrupted using site‐directed mutagenesis.

## Materials and methods

### Chemicals

Blend Taq^®^ and *Taq* DNA polymerase were purchased from TOYOBO (Osaka, Japan) and New England Biolabs (Ipswich, MA, USA), respectively. T4 DNA Ligase was purchased from Takara Bio (Kusatsu, Japan). Restriction enzymes were purchased from Takara Bio and TOYOBO. Other general reagents were purchased from Wako Pure Chemical Industries (Osaka, Japan) and Nacalai Tesque (Kyoto, Japan).

### Protein manipulation

Visualization of molecular structures was carried out using the Accelrys Discovery Studio Visualizer 4.0 (BIOVIA, Dassault Systèmes, San Diego, CA, USA). Atomic coordinate data—1YSC, 3SC2, 1IVY, and 4MWS—were obtained from the RCSB Protein Data Bank (http://www.rcsb.org) for mCPY, CPWII, precursor PPCA, and mature PPCA, respectively.

### Plasmid construction

Sequence data of CPY, *prc1*/YMR297W (SGD ID: SGD: S000004912), were obtained from the *Saccharomyces* Genome Database (http://www.yeastgenome.org/). The recombinant wild‐type CPY expression vector was prepared as described 14: The *prc1* fragment encoding full‐length wild‐type CPY was prepared from yeast genomic DNA extracted from yeast wild‐type strain using PCR amplification with primers containing a *Sma*I or *Xho*I restriction site for the 5′‐ and 3′‐termini, respectively. This fragment was inserted into the pGEM^®^‐T Easy Vector (Promega, Madison, WI, USA) to construct the cloning vector, which was digested with *Sma*I and *Xho*I to generate an insert fragment. Separately, the yeast expression vector, pLTex321sV5H 15, was digested with *Sma*I and* Xho*I to generate the template fragment. The two fragments were ligated to construct the recombinant wild‐type CPY expression vector.

Plasmid vectors for mutant CPYs—C193A, C207A, C262A, and C268A—were constructed using the QuikChange site‐directed mutagenesis method (Agilent Technologies, Santa Clara, CA, USA). This method utilized the constructed recombinant wild‐type CPY expression vector as a template with the following oligonucleotide primer pairs:

C193A‐F: 5′‐GAACCAATGGCCGCTGGTGAAGGTG‐3′
C193A‐R: 5′‐CACCTTCACCAGCGGCCATTGGTTC‐3′
C207A‐F: 5′‐CCCTCGGAGGAAGCCTCTGCTATGGAA‐3′
C207A‐R: 5′‐TTCCATAGCAGAGGCTTCCTCCGAGGG‐3′
C262A‐F: 5′‐CAGGAAGGATGCTGAAGGTGGCAA‐3′
C262A‐R: 5′‐TTGCCACCTTCAGCATCCTTCCTG‐3′
C268A‐F: 5′‐GGTGGCAATTTGGCCTACCCAACGT‐3′
C268A‐R: 5′‐ACGTTGGGTAGGCCAAATTGCCACC‐3′

The double‐underlines denote the mutation sites. All the primers were synthesized by Eurofins Genomics (Tokyo, Japan). Each mutation introduced into the target site was confirmed by DNA sequencing that was performed at Fasmac (Atsugi, Japan).

### Expression and purification of recombinant wild‐type and mutant CPYs

All mutants were expressed and purified by the same method for the recombinant wild‐type CPY preparation. *Saccharomyces cerevisiae* BY4741*prc1Δ* (*MATa*,* his3Δ1*,* leu2Δ0*,* met15Δ0*,* ura3Δ0*, and* prc1Δ::kanMX4*) purchased from Thermo Fisher Scientific (Waltham, MA, USA) was transformed with the constructed plasmid vector using the lithium acetate method 16. Transformants were selected on SD‐Ura agar plate containing 0.67% (w/v) yeast nitrogen base without amino acids, 2% (w/v) glucose, 2% (w/v) agar, 30 μg·mL^−1^ l‐leucine, 20 μg·mL^−1^ l‐histidine monohydrochloride monohydrate, 30 μg·mL^−1^ adenine hemisulfate dihydrate, and 20 μg·mL^−1^ l‐methionine. The selected yeast cells were grown by shaking in YPD liquid medium containing 2% (w/v) polypeptone, 1% (w/v) yeast extract, and 2% (w/v) glucose at 28 °C until the optical density at 600 nm exceeded 1.0. The culture was incubated for an additional 72 h at 20 °C to induce the expression of recombinant CPY. Cells were harvested by centrifugation at 2000 ***g***, and the pellet was stored at −30 °C until used.

Purification of each CPY was performed as described previously 17. The harvested cells were autolyzed with chloroform and water and centrifuged. The supernatant was collected, ammonium sulfate fractionation was carried out at 20%, and precipitates were removed. A second ammonium sulfate fractionation was carried out to 90%, and the precipitated proteins were collected. The precipitate was dissolved in 50 mm sodium acetate buffer, pH 5.0, and incubated at room temperature for 18 h so that overexpressed CPY protein was completely matured. After dialysis against 10 mm sodium phosphate buffer at pH 7.0, mCPY was purified by TOYOPEARL^®^ Butyl‐650M column chromatography (1.5 cm × 5 cm; Tosoh, Tokyo, Japan).

Each cell that expressed C193A or C207A mutant protein was also homogenized using Mini‐Beadbeater (BioSpec Products, Bartlesville, OK, USA) to confirm the influence of the method of cell lysis for aggregation.

### SDS/PAGE and western blotting

Expression and purification of the recombinant CPYs were confirmed by SDS/PAGE and western blotting. The rabbit serum containing polyclonal anti‐CPY antibody (1 : 7500) was prepared in our laboratory. Horseradish peroxidase‐conjugated anti‐rabbit IgG secondary antibody (1 : 100 000) was purchased from GE Healthcare (Buckinghamshire, UK).

### Protein assay

The protein concentration of each solution in the extraction and purification steps was estimated using the Quick Start Bradford Protein Assay Kit (Bio‐Rad, Hercules, CA, USA) using bovine serum albumin as the standard protein. After the color development, protein contents were determined photometrically.

### Activity assays

Peptidase activity of each purified recombinant CPY was assayed by incubating with *N*‐benzyloxycarbonyl‐l‐phenylalanyl‐l‐leucine (Z‐Phe‐Leu‐OH; Bachem, Bubendorf, Switzerland) at pH 7.0 and room temperature. l‐Leucine, a hydrolysis product, was measured by amino acid analysis using the LaChromUltra U‐HPLC system (Hitachi High‐Technologies, Tokyo, Japan). Anilidase activity of each CPY was determined spectrophotometrically 17. The release of *p*‐nitroaniline from *N*‐benzoyl‐l‐tyrosine *p*‐nitroanilide (Bz‐Tyr‐*p*NA; Sigma‐Aldrich, St. Louis, MO, USA) at pH 7.0 and room temperature was monitored at 410 nm using a model U‐2000A spectrophotometer (Hitachi High‐Technologies).

## Results

### Expression patterns of recombinant mutant CPYs

**Figure 2:**
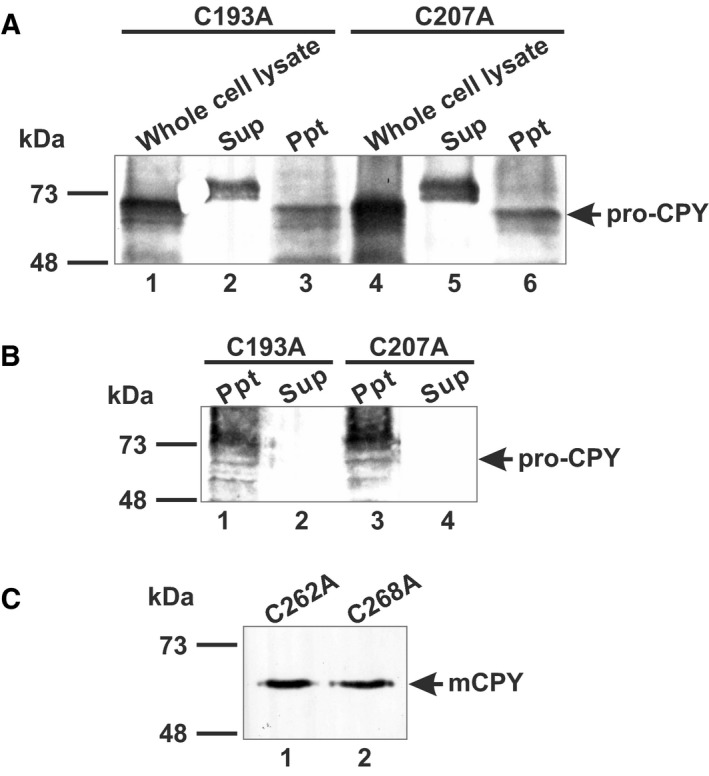
Expression of cysteine‐substituted mutants in yeast cells. Following inductive cultivation, yeast cells were treated with chloroform and water for autolysis and the soluble fraction was subjected to ammonium sulfate precipitation and hydrophobic interaction chromatography. (A) Whole‐cell lysate (lane 1, 4), soluble fraction (Sup; lane 2, 5), and precipitates (Ppt; lane 3, 6) after autolysis, for C193A and C207A, respectively. Samples were analyzed by western blotting using anti‐CPY antiserum diluted 1 : 7500. The arrow indicates the migration position of a precursor CPY (pro‐CPY). (B) Western blot analysis of precipitates (Ppt; lane 1, 3) and soluble fraction (Sup; lane 2, 4) obtained after bead beating homogenization of yeast cells containing C193A or C207A, respectively. Anti‐CPY antiserum (1 : 7500) was used. Lanes 1 and 2 represent C193A, and lanes 3 and 4 represent C207A. The arrow indicates the migration position of pro‐CPY. (C) Western blot analysis of C262A and C268A purified by hydrophobic interaction chromatography. Anti‐CPY antiserum (1 : 7500) was used. Lane 1 contains C262A, and lane 2 contains C268A. The arrow indicates the migration position of mCPY.

Expression of each recombinant mutant CPY was confirmed. After the inducible cultivation, yeast cells were directly lysed in the SDS/PAGE sample buffer and analyzed by western blotting with anti‐CPY antiserum. Western blotting revealed that each mutant CPY was expressed in the yeast cells as a 69‐kDa precursor form similar to the wild‐type CPY (data not shown). As this analysis did not provide information about protein folding, solubility was examined after autolysis by chloroform and water. C193A and C207A were recovered in the insoluble fraction, while C262A and C268A were in the soluble fraction (Fig. 2A,C). We also employed the bead beater method of homogenization for C193A and C207A and detected both mutant proteins in the precipitates using western blotting (Fig. 2B).

As SDS/PAGE and western blot analyses of the solubilized whole‐cell lysates detected the main band at ~ 69 kDa, it is likely that the recombinant protein was expressed. Both C193A and C207A mutants were found in the insoluble fraction after autolysis or bead beater treatment. Thus, these mutant proteins were likely aggregated during the protein extraction process. As the C193A and C207A mutants were unable to form disulfide bonds, some fluctuation would be introduced at the V‐shape helix. This conformational instability might be the reason for the insoluble behavior during cell disruption and/or protein folding. C262A and C268A were purified as soluble proteins (Fig. 2C). The collective findings suggest that the disulfide bond at C193–C207 could be related to maintenance of the structure of the V‐shape helix.

### Hydrolase activity of mutant CPYs

Catalytic activity is an excellent index for the structural change occurring in CPYs 18. The importance of disulfide bonds to the V‐shape helix was evaluated by measuring enzyme activity. As C193A and C207A proteins failed to yield a soluble enzyme, we only examined C262A and C268A. Hydrolase activity was measured using synthetic substrates, where peptidase activity was determined by measuring the release rate of l‐leucine from Z‐Phe‐Leu‐OH, and anilidase activity was determined by measuring the release rate of *p*‐nitroaniline from Bz‐Tyr‐*p*NA. Table 1 summarizes the hydrolase activities for wild‐type CPY, C262A, and C268A. When peptidase activities are compared between mutant and wild‐type, C262A and C268A reduced activities to 8.5% and 16% that of the wild‐type, respectively. Similarly, anilidase activities were reduced to 1.8% and 1.9%, respectively. The results indicate that the disruption of C262–C268 affects the catalytic activity of CPY, where anilidase activity is more susceptible than peptidase activity.

**Table 1:** Relative specific activity of wild‐type and soluble mutant CPYs toward Z‐Phe‐Leu‐OH and Bz‐Tyr‐*p*NA

CPY	Z‐Phe‐Leu‐OH^a^	Bz‐Tyr‐*p*NA^b^
WT	(100)	(100)
C262A	8.5	1.8
C268A	16	1.9

a Enzyme reaction was performed with 1 mm Z‐Phe‐Leu‐OH at pH 7.0 and room temperature. The amount of released l‐leucine was determined by U‐HPLC.

b Enzyme reaction was performed with 0.3 mm Bz‐Tyr‐*p*NA at pH 7.0 and room temperature, and an absorbance at 410 nm was monitored spectrophotometrically.

## Discussion

### Contribution of the V‐shape helix to CPY catalysis

The insertion domain in lipases and acetylcholinesterases, which is present in most members of the α/β hydrolase fold family, has a unique catalytic role 7, 8, 9, 10. CPY and related serine carboxypeptidases, which belong to this α/β hydrolase fold family, have a corresponding helical insertion domain, although the involvement of insertion domain in the catalytic function is not clearly defined. This study intended to reveal a possible role(s) of the insertion domain in CPY by disrupting two sets of disulfide bonds that support the helical structure (the V‐shape helix) in the insertion domain. When the disulfide bond of C262–C268 was disrupted, the mutant enzyme lost both peptidase and anilidase activities (Table 1). A C262–C268 bridge located on the flexible loop at the exit side of the V‐shape helix may contribute significantly to the catalytic process, probably by stabilizing the V‐shape helix from unnecessary fluctuation during the catalysis.

The substrate‐binding sites, S_1_′ and S_1_–S_5_, and the hydrogen bond network located at the bottom of the S_1_′ site participate in substrate recognition 19, 20, 21, 22, 23. Indeed, some amino acid residues at the V‐shape helix comprise part of those substrate‐binding sites. Our previous findings support the idea that the V‐shape helix participates in the catalysis because the replacement of the residues at or near the V‐shape helix influences the hydrolase activity of CPY 14. To further elucidate this function of the V‐shape helix, dynamic analyses, such as molecular dynamic simulations or kinetic analyses, would be required. It is important to note that a disulfide bridge equivalent to C262–C268 is present in both CPWII and PPCA. CPY and other serine carboxypeptidases differ slightly, in that the loop region in CPY bridged by the disulfide bond is short, whereas in CPWII and PPCA, the same region is long and a portion can be cleaved during maturation 12, 24. Therefore, it seems likely that the V‐shape helix is necessary not only for catalysis, but also to maintain the active mature form, especially in the presence of C262–C268 or a corresponding bond.

### Relationship between the V‐shape helix and structural stability in CPY

In some cases, disulfide bond(s) help maintain protein conformation. Any disulfide bond disruption eventually leads to destabilization of protein structure. In yeast, such destabilized proteins are eliminated by the endoplasmic reticulum‐associated degradation (ERAD) pathway, which efficiently recognizes and disposes of abnormal proteins 25. One well‐known example is a mutated CPY 26, 27. If recombinant CPYs are unfolded or misfolded in the ER, they become a target for ERAD. Similarly, mutant CPYs with disrupted disulfide zipper at C217–C240 and C224–C233 are susceptible for proteolysis 11. As shown in Fig. 2A, recombinant C193A and C207A mutants were insoluble, but were not fragmented in yeast host cells. This indicates that the mutants were not a target of the ERAD pathway. The maintenance of the V‐shape helix by the C193–C207 disulfide bridge may be important for the stable preservation of the folded CPY structure.

**Figure 3:**
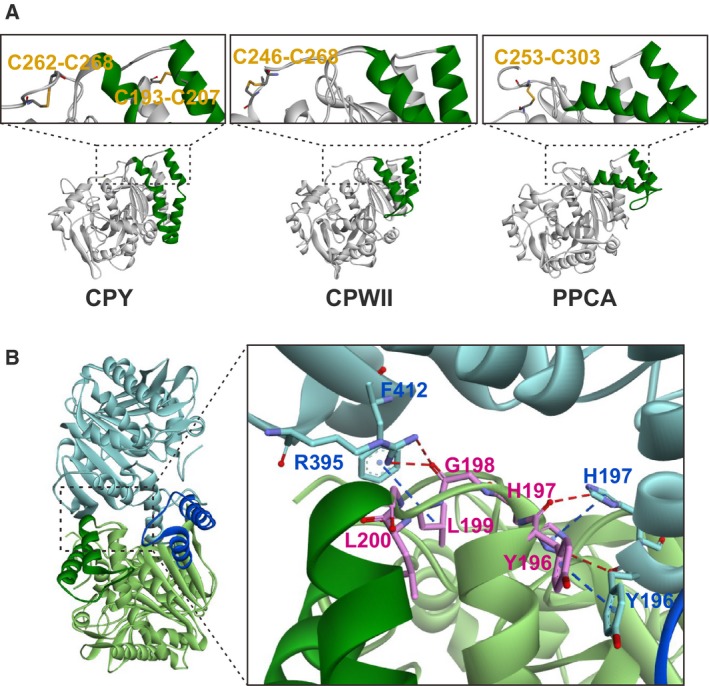
Three‐dimensional structural comparison of serine carboxypeptidases: CPY, CPWII (PDB ID: 3SC2 12), and PPCA (PDB ID: 4MWS 24). (A) Close‐up view of the beginning and end of the V‐shape helix or corresponding helical region of CPY, CPWII, and PPCA. The V‐shape helix of CPY and corresponding helical regions of the other two enzymes are colored green. C193–C207 is a CPY‐specific disulfide bond, and disulfide bonds corresponding to C262–C268 of CPY are identified in CPWII and PPCA. (B) Homodimer structure and the dimer interface of PPCA. Each core domain is colored light green and light blue, and the helical region is colored green and blue. In PPCA, the disulfide bond corresponding C193–C207 of CPY was not formed in the loop region at the beginning of the helical region. The residues of this loop region (196–200, colored pink) interact with the residues of paired subunit (colored light blue) by forming hydrogen bonds (red broken line) and hydrophobic interaction (blue broken line). Those intramolecular interactions were monitored using Accelrys Discovery Studio Visualizer 4.0.

A comparison between the structures of CPY and other homologous serine carboxypeptidases CPWII and PPCA did not reveal a disulfide bond corresponding to C193–C207 in CPY (Fig. 3A). As C193–C207 contributes to the stability of CPY, there should be a different mechanism to replace the role of C193–C207 in both CPWII and PPCA. A hint to this mechanism is that both CPWII and PPCA are homodimers 12, 13, 24. Two subunits are associated with each other around the insertion domain where the loop region is included at this interface (Fig. 3B). It seems probable that either the C193–C207 disulfide bridge or dimer formation contributes to a flexible loop structure at the proper place. However, the details remain to be determined.

The present results demonstrate that the V‐shape helix in CPY is involved in its catalysis and maintenance of conformation. These findings can be extended to other serine carboxypeptidases belonging to the α/β hydrolase fold family. The insertion domain of this family is quite diverse. This diversity is reflected in the wide range of catalytic properties that have been observed for serine proteases 6, 28. We expect that further studies on the insertion domains will unravel the mystery of evolution and differentiation of α/β hydrolase fold family enzymes.

## Conflict of interest

The authors declare no conflict of interest.

## Author contributions

HU conceived and directed the study. MM designed and performed the experiments. TS and NM provided the yeast expression vectors. MM wrote the manuscript. TS, NM, and HU revised the manuscript.
